# 1,15:21,35-Bis(oxydiethyl­ene)-5,8,11,18,25,28,31,38-octa­oxa-1,15,21,35-tetra­azacyclo­tetra­decane-16,20,36,40-tetra­one–benzene (1/2): a macrotricyclic tetra­lactam

**DOI:** 10.1107/S1600536809026531

**Published:** 2009-07-18

**Authors:** Gary L. N. Smith, Tam Nguyen, Douglas R. Powell, Richard W. Taylor

**Affiliations:** a Point Loma Nazarene University, Department of Chemistry, 3900 Lomaland Dr., Rohr Science 305E, San Diego, CA 92106, USA; b Department of Chemistry and Biochemistry, University of Oklahoma, 620 Parrington Oval, Room 208, Norman, OK 73019-3051, USA

## Abstract

The macrotricyclic title compound, C_36_H_64_N_4_O_14_·2C_6_H_6_, is located on a crystallographic center of symmetry. The mol­ecule has four tertiary amide bridgehead atoms and consists of two unsymmetrical 20-membered diaza­tetra­oxamacrocycles (N_2_O_4_ donor atom set) connected through the N atoms by two lateral oxydiethyl­ene bridges. The bridging subunits, together with the short bridging strand (NCCOCCN) from each monocycle, define a 24-membered ring (N_4_O_4_ donor atom set) that forms a central cavity.

## Related literature

For general background to macrotricyclic ligands as receptors for cationic, anionic and neutral guests, see: Lehn (1973 ▶, 1988 ▶); Lehn *et al.* (1977 ▶). For related structures, see: Wiest & Weiss (1973 ▶); Fischer *et al.* (1977 ▶); Pascard *et al.* (1982 ▶); Rebizant *et al.* (1984 ▶); Groth (1986 ▶); Cheetham & Harding (1991 ▶); Bencini *et al.* (1992 ▶); Krakowiak *et al.* (1995 ▶); Plenio & Diodone (1995 ▶); Smith *et al.* (2007 ▶). For the synthesis, see: Dietrich *et al.* (1973 ▶); Cheney *et al.* (1978 ▶).
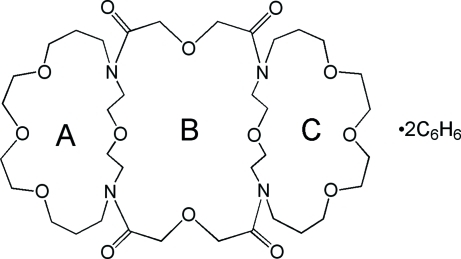


## Experimental

### 

#### Crystal data

C_36_H_64_N_4_O_14_·2C_6_H_6_
*M* *_r_* = 933.13
Triclinic, 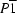

*a* = 8.9632 (14) Å
*b* = 11.988 (2) Å
*c* = 12.806 (2) Å
α = 72.728 (5)°
β = 71.758 (5)°
γ = 68.443 (6)°
*V* = 1189.4 (3) Å^3^
*Z* = 1
Mo *K*α radiation
μ = 0.10 mm^−1^
*T* = 100 K
0.41 × 0.37 × 0.30 mm

#### Data collection

Bruker APEX CCD diffractometer
Absorption correction: multi-scan (*SADABS*; Sheldrick, 2007 ▶) *T*_min_ = 0.954, *T*_max_ = 0.978
9363 measured reflections
4603 independent reflections
4198 reflections with *I* > 2σ(*I*)
*R*_int_ = 0.018

#### Refinement

*R*[*F* ^2^ > 2σ(*F* ^2^)] = 0.035
*wR*(*F* ^2^) = 0.089
*S* = 1.00
4603 reflections
299 parameters
H-atom parameters constrained
Δρ_max_ = 0.27 e Å^−3^
Δρ_min_ = −0.21 e Å^−3^

### 

Data collection: *SMART* (Bruker, 1998 ▶); cell refinement: *SAINT* (Bruker, 1998 ▶); data reduction: *SAINT*; program(s) used to solve structure: *SHELXTL* (Sheldrick, 2008 ▶); program(s) used to refine structure: *SHELXTL*; molecular graphics: *SHELXTL*; software used to prepare material for publication: *SHELXTL*.

## Supplementary Material

Crystal structure: contains datablocks I, global. DOI: 10.1107/S1600536809026531/pk2175sup1.cif

Structure factors: contains datablocks I. DOI: 10.1107/S1600536809026531/pk2175Isup2.hkl

Additional supplementary materials:  crystallographic information; 3D view; checkCIF report

## Figures and Tables

**Fig. 1.**
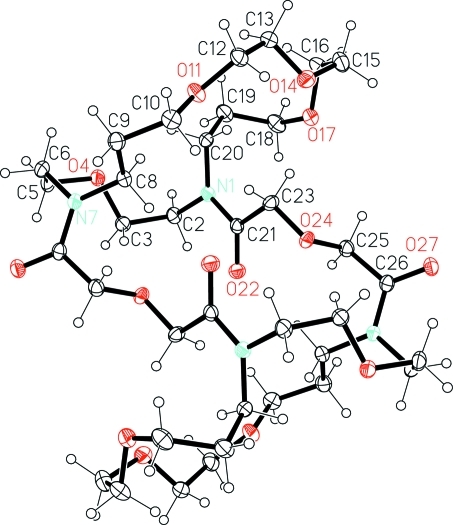
The molecular structure of (I), showing the atom-numbering scheme. Displacement ellipsoids are drawn at the 50% probability level. Unlabelled atoms are related to their labelled counterparts by (1-x, 1-y, 1-z).

**Fig. 2.**
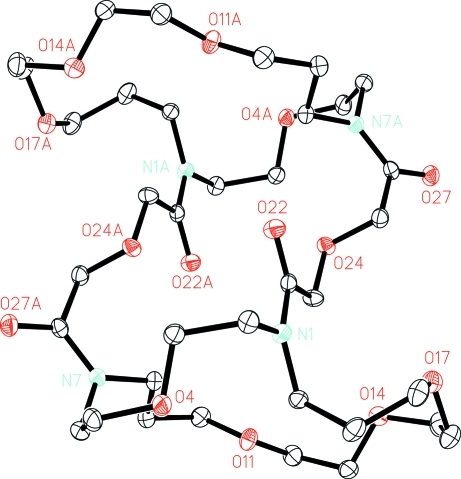
A view of the molecular structure of (I), along an axis perpendicular to the plane defined by the donor atoms of the 24-membered ring (B). Atoms marked with the letter A are related to unsubscripted atoms by (1-x, 1-y, 1-z). H atoms have been omitted for clarity.

## supplementary crystallographic information

### Comment

Macrotricyclic ligands are of interest as receptors for cationic, anionic and neutral guests (Lehn *et al.*, 1977; Lehn, 1988). The title compound (I) was isolated as the 2 + 2 addition product during the synthesis of the corresponding bicyclic cryptand 3pp1.1. A related macrotricyclic tetraamine has been reported, but with the propylene groups in (I) replaced by benzene rings (Smith *et al.*, 2007). Fig. 1 shows that (I) contains three monocyclic rings: a 24-membered ring, B (N1/O4/N7/O24A/N1A/O4A/N7A/O24) and two 20-membered rings, A (N1/O4/N7/O11/O14/O17) and C (N1A/O4A/N7A/O11A/O14A/O17A). The two 20-membered macrocyclic rings form the ends of the skewed cylinder with two oxydiethylene bridges linking the macrocylic end groups. Each of the 20-membered rings (A and C) has an elliptical shape, with non-bonding distances of 4.495 (2)Å (N1···O11), 6.639 (2)Å (O4···O14), and 7.209 (2)Å (N7···O17). The planes defined by donor atoms (N1/N7/O11/O14/O17) of rings A (and C) (average deviation = 0.1796 Å) are parallel, and form a dihedral angle of 100.7 (2)° with the plane defined by the nitrogen donor atoms in ring B. Thus, the centers of rings A and C do not overlap in the direction defined by the O4···O14 axis. The two 20-membered rings are oriented in an opposing fashion with respect to the plane defined by the N donor atoms in ring B. Similar behavior has been seen with other cylindrical tricyclic cryptands having unsymmetric end groups. (Groth, 1986; Cheetham & Harding, 1991; Plenio & Diodone, 1995). Fig. 2 shows that the nitrogen atoms in the 24-membered ring form the corners of a parallelogram defined by the following non-bonding distances and angles: 4.300 (2)Å (N1···N7); 6.901 (2)Å (N7···N1A); 74.0 (2)° (N1···N7···N1A); 106.0 (2)° (N7···N1···N7A). As a result, the 20-membered rings are also offset along the O24···O24A axis. Analogous macrotricyclic compounds also exhibit a skewed cylindrical shape (Bencini *et al.*, 1992; Pascard *et al.*, 1982; Rebizant *et al.*, 1984: Smith *et al.*, 2007).

### Experimental

The 20-membered monocyclic diamine was prepared according to reported methods (Dietrich *et al.*, 1973). The tricyclic tetralactam was obtained as the 2 + 2 cycloaddition product from the reaction of 1,7-diaza-4,11,14,17-tetraoxacycloicosane (3.56 mmole) in 150 ml toluene and 2,2'-oxydiacetyl chloride (3.54 mmole) in 150 ml toluene. These solutions were added synchronously to 800 ml of toluene containing triethylamine (7.80 mmole) over a period of 2 h under high-dilution conditions (Dietrich *et al.*, 1973; Cheney *et al.*, 1978). The crude tetralactam was purified by recrystallization from benzene to give (I) in 10% overall yield. ESI-MS: m/*z* = 799.5 (*M* + Na^+^). Crystals suitable for X-ray crystallography were grown by vapor diffusion of heptane into a solution of (I) in benzene.

### Refinement

H atoms were positioned geometrically and refined using a riding model with C—H = 0.99Å for RCH_2_R and 0.95Å for H atoms in the aromatic solvent; *U*_iso_(H) = 1.2 *U*_eq_(C).

### Crystal data

**Table:**

C_36_H_64_N_4_O_14_·2C_6_H_6_	*Z* = 1
*M**_r_* = 933.13	*F*(000) = 504
Triclinic, *P*1	*D*_x_ = 1.303 Mg m^−^^3^
Hall symbol: -P 1	Mo *K*α radiation, λ = 0.71073 Å
*a* = 8.9632 (14) Å	Cell parameters from 7833 reflections
*b* = 11.988 (2) Å	θ = 2.3–28.3°
*c* = 12.806 (2) Å	µ = 0.10 mm^−^^1^
α = 72.728 (5)°	*T* = 100 K
β = 71.758 (5)°	Block, colorless
γ = 68.443 (6)°	0.41 × 0.37 × 0.30 mm
*V* = 1189.4 (3) Å^3^	

### Data collection

**Table:**

Bruker APEX CCD diffractometer	4603 independent reflections
Radiation source: fine-focus sealed tube	4198 reflections with *I* > 2σ(*I*)
graphite	*R*_int_ = 0.018
ω scans	θ_max_ = 26.0°, θ_min_ = 2.3°
Absorption correction: multi-scan (*SADABS*; Sheldrick, 2007)	*h* = −11→11
*T*_min_ = 0.954, *T*_max_ = 0.978	*k* = −14→13
9363 measured reflections	*l* = −15→15

### Refinement

**Table:**

Refinement on *F*^2^	Secondary atom site location: difference Fourier map
Least-squares matrix: full	Hydrogen site location: inferred from neighbouring sites
*R*[*F*^2^ > 2σ(*F*^2^)] = 0.035	H-atom parameters constrained
*wR*(*F*^2^) = 0.089	*w* = 1/[σ^2^(*F*_o_^2^) + (0.04*P*)^2^ + 0.42*P*] where *P* = (*F*_o_^2^ + 2*F*_c_^2^)/3
*S* = 1.00	(Δ/σ)_max_ = 0.001
4603 reflections	Δρ_max_ = 0.27 e Å^−^^3^
299 parameters	Δρ_min_ = −0.21 e Å^−^^3^
0 restraints	Extinction correction: *SHELXTL* (Sheldrick, 2008), Fc^*^=kFc[1+0.001xFc^2^λ^3^/sin(2θ)]^-1/4^
Primary atom site location: structure-invariant direct methods	Extinction coefficient: 0.0145 (16)

### Fractional atomic coordinates and isotropic or equivalent isotropic displacement parameters (Å^2^)

**Table:**

	*x*	*y*	*z*	*U*_iso_*/*U*_eq_	
N1	0.47767 (12)	0.74664 (8)	0.56224 (8)	0.0167 (2)	
C2	0.53952 (14)	0.83074 (10)	0.46324 (9)	0.0186 (2)	
H2A	0.6567	0.7900	0.4317	0.022*	
H2B	0.5341	0.9039	0.4868	0.022*	
C3	0.44488 (14)	0.87172 (10)	0.37224 (9)	0.0188 (2)	
H3A	0.5076	0.9109	0.3015	0.023*	
H3B	0.4303	0.7998	0.3585	0.023*	
O4	0.28675 (10)	0.95714 (7)	0.40616 (7)	0.02112 (19)	
C5	0.18854 (16)	0.99679 (11)	0.32485 (10)	0.0224 (3)	
H5A	0.2614	0.9827	0.2508	0.027*	
H5B	0.1317	1.0859	0.3176	0.027*	
C6	0.06115 (15)	0.92948 (11)	0.35714 (10)	0.0217 (3)	
H6A	−0.0156	0.9479	0.4289	0.026*	
H6B	−0.0037	0.9605	0.2989	0.026*	
N7	0.13369 (12)	0.79611 (9)	0.36959 (8)	0.0176 (2)	
C8	0.12705 (14)	0.72108 (11)	0.48393 (9)	0.0179 (2)	
H8A	0.1507	0.7626	0.5304	0.021*	
H8B	0.2139	0.6412	0.4809	0.021*	
C9	−0.03960 (14)	0.69865 (11)	0.53976 (9)	0.0208 (3)	
H9A	−0.1261	0.7780	0.5470	0.025*	
H9B	−0.0663	0.6605	0.4919	0.025*	
C10	−0.03863 (15)	0.61574 (11)	0.65499 (10)	0.0215 (3)	
H10A	−0.1487	0.6043	0.6904	0.026*	
H10B	0.0429	0.5344	0.6479	0.026*	
O11	0.00290 (10)	0.66990 (7)	0.72305 (6)	0.02062 (19)	
C12	−0.04284 (15)	0.61758 (12)	0.83951 (9)	0.0228 (3)	
H12A	−0.0026	0.5271	0.8509	0.027*	
H12B	−0.1645	0.6429	0.8651	0.027*	
C13	0.02910 (15)	0.65877 (12)	0.90752 (10)	0.0236 (3)	
H13A	0.0266	0.7453	0.8757	0.028*	
H13B	−0.0368	0.6529	0.9860	0.028*	
O14	0.19488 (10)	0.58292 (8)	0.90553 (7)	0.0235 (2)	
C15	0.26383 (16)	0.59898 (12)	0.98450 (10)	0.0256 (3)	
H15A	0.3544	0.5238	1.0025	0.031*	
H15B	0.1782	0.6097	1.0548	0.031*	
C16	0.32977 (16)	0.70713 (12)	0.94297 (10)	0.0254 (3)	
H16A	0.2425	0.7826	0.9204	0.031*	
H16B	0.3661	0.7186	1.0035	0.031*	
O17	0.46530 (10)	0.68442 (8)	0.84922 (7)	0.0226 (2)	
C18	0.50076 (16)	0.79286 (12)	0.77925 (10)	0.0247 (3)	
H18A	0.6099	0.7698	0.7272	0.030*	
H18B	0.5070	0.8421	0.8267	0.030*	
C19	0.37289 (16)	0.87074 (11)	0.71125 (10)	0.0235 (3)	
H19A	0.4124	0.9382	0.6575	0.028*	
H19B	0.2697	0.9081	0.7628	0.028*	
C20	0.33413 (14)	0.80088 (10)	0.64589 (9)	0.0177 (2)	
H20A	0.2915	0.7348	0.6996	0.021*	
H20B	0.2461	0.8576	0.6072	0.021*	
C21	0.54820 (14)	0.62431 (10)	0.56542 (9)	0.0159 (2)	
O22	0.66849 (10)	0.58351 (7)	0.49363 (6)	0.01991 (19)	
C23	0.46919 (14)	0.53806 (10)	0.66279 (9)	0.0180 (2)	
H23A	0.4537	0.5610	0.7345	0.022*	
H23B	0.3593	0.5472	0.6537	0.022*	
O24	0.56807 (9)	0.41414 (7)	0.66696 (6)	0.01685 (18)	
C25	0.70820 (14)	0.38705 (10)	0.71013 (9)	0.0184 (2)	
H25A	0.6741	0.4119	0.7833	0.022*	
H25B	0.7847	0.4319	0.6574	0.022*	
C26	0.79233 (13)	0.24948 (10)	0.72452 (9)	0.0166 (2)	
O27	0.79022 (10)	0.18395 (7)	0.81918 (6)	0.02115 (19)	
C1S	0.23336 (16)	0.07749 (12)	0.97250 (11)	0.0275 (3)	
H1S	0.2265	0.0058	1.0285	0.033*	
C2S	0.13507 (16)	0.12252 (12)	0.89521 (10)	0.0258 (3)	
H2S	0.0591	0.0824	0.8989	0.031*	
C3S	0.14782 (15)	0.22620 (12)	0.81243 (10)	0.0253 (3)	
H3S	0.0809	0.2566	0.7593	0.030*	
C4S	0.25741 (15)	0.28561 (12)	0.80677 (10)	0.0252 (3)	
H4S	0.2665	0.3560	0.7495	0.030*	
C5S	0.35382 (15)	0.24199 (12)	0.88490 (11)	0.0257 (3)	
H5S	0.4279	0.2833	0.8820	0.031*	
C6S	0.34207 (16)	0.13823 (12)	0.96721 (11)	0.0273 (3)	
H6S	0.4087	0.1083	1.0204	0.033*	

### Atomic displacement parameters (Å^2^)

**Table:**

	*U*^11^	*U*^22^	*U*^33^	*U*^12^	*U*^13^	*U*^23^
N1	0.0211 (5)	0.0133 (5)	0.0153 (4)	−0.0056 (4)	−0.0043 (4)	−0.0019 (4)
C2	0.0237 (6)	0.0147 (5)	0.0178 (5)	−0.0080 (5)	−0.0051 (4)	−0.0009 (4)
C3	0.0229 (6)	0.0154 (5)	0.0176 (5)	−0.0050 (5)	−0.0048 (4)	−0.0034 (4)
O4	0.0265 (4)	0.0169 (4)	0.0188 (4)	−0.0007 (3)	−0.0096 (3)	−0.0049 (3)
C5	0.0309 (7)	0.0144 (6)	0.0202 (6)	−0.0014 (5)	−0.0122 (5)	−0.0013 (4)
C6	0.0238 (6)	0.0173 (6)	0.0207 (6)	0.0020 (5)	−0.0092 (5)	−0.0054 (5)
N7	0.0191 (5)	0.0153 (5)	0.0165 (5)	−0.0014 (4)	−0.0066 (4)	−0.0027 (4)
C8	0.0190 (6)	0.0196 (6)	0.0149 (5)	−0.0045 (5)	−0.0059 (4)	−0.0030 (4)
C9	0.0199 (6)	0.0238 (6)	0.0203 (6)	−0.0058 (5)	−0.0070 (5)	−0.0056 (5)
C10	0.0228 (6)	0.0219 (6)	0.0224 (6)	−0.0096 (5)	−0.0046 (5)	−0.0053 (5)
O11	0.0266 (4)	0.0225 (4)	0.0148 (4)	−0.0122 (4)	−0.0041 (3)	−0.0017 (3)
C12	0.0212 (6)	0.0262 (6)	0.0169 (6)	−0.0085 (5)	−0.0020 (4)	0.0006 (5)
C13	0.0221 (6)	0.0262 (6)	0.0168 (5)	−0.0020 (5)	−0.0028 (5)	−0.0047 (5)
O14	0.0207 (4)	0.0255 (5)	0.0236 (4)	−0.0031 (4)	−0.0064 (3)	−0.0075 (4)
C15	0.0279 (7)	0.0303 (7)	0.0184 (6)	−0.0102 (5)	−0.0079 (5)	−0.0005 (5)
C16	0.0303 (7)	0.0304 (7)	0.0164 (6)	−0.0111 (5)	−0.0030 (5)	−0.0062 (5)
O17	0.0236 (4)	0.0240 (5)	0.0183 (4)	−0.0061 (4)	−0.0051 (3)	−0.0026 (3)
C18	0.0294 (7)	0.0307 (7)	0.0198 (6)	−0.0161 (5)	−0.0047 (5)	−0.0056 (5)
C19	0.0367 (7)	0.0159 (6)	0.0181 (6)	−0.0090 (5)	−0.0057 (5)	−0.0034 (4)
C20	0.0205 (6)	0.0139 (5)	0.0165 (5)	−0.0020 (4)	−0.0050 (4)	−0.0031 (4)
C21	0.0190 (6)	0.0151 (5)	0.0154 (5)	−0.0044 (4)	−0.0071 (4)	−0.0031 (4)
O22	0.0209 (4)	0.0173 (4)	0.0186 (4)	−0.0047 (3)	−0.0017 (3)	−0.0038 (3)
C23	0.0179 (6)	0.0128 (5)	0.0200 (5)	−0.0026 (4)	−0.0027 (4)	−0.0032 (4)
O24	0.0179 (4)	0.0119 (4)	0.0206 (4)	−0.0031 (3)	−0.0066 (3)	−0.0027 (3)
C25	0.0217 (6)	0.0172 (6)	0.0181 (5)	−0.0054 (5)	−0.0076 (4)	−0.0039 (4)
C26	0.0160 (5)	0.0174 (6)	0.0185 (5)	−0.0051 (4)	−0.0074 (4)	−0.0028 (4)
O27	0.0256 (4)	0.0196 (4)	0.0169 (4)	−0.0050 (3)	−0.0081 (3)	−0.0009 (3)
C1S	0.0335 (7)	0.0196 (6)	0.0252 (6)	−0.0047 (5)	−0.0063 (5)	−0.0032 (5)
C2S	0.0256 (6)	0.0269 (7)	0.0273 (6)	−0.0102 (5)	−0.0033 (5)	−0.0092 (5)
C3S	0.0212 (6)	0.0309 (7)	0.0215 (6)	−0.0039 (5)	−0.0061 (5)	−0.0060 (5)
C4S	0.0230 (6)	0.0242 (6)	0.0227 (6)	−0.0059 (5)	−0.0008 (5)	−0.0029 (5)
C5S	0.0193 (6)	0.0302 (7)	0.0291 (6)	−0.0077 (5)	−0.0013 (5)	−0.0125 (5)
C6S	0.0246 (6)	0.0299 (7)	0.0256 (6)	0.0006 (5)	−0.0104 (5)	−0.0096 (5)

### Geometric parameters (Å, °)

**Table:**

N1—C21	1.3606 (15)	C15—H15B	0.9900
N1—C2	1.4639 (14)	C16—O17	1.4245 (14)
N1—C20	1.4702 (14)	C16—H16A	0.9900
C2—C3	1.5137 (15)	C16—H16B	0.9900
C2—H2A	0.9900	O17—C18	1.4240 (14)
C2—H2B	0.9900	C18—C19	1.5163 (18)
C3—O4	1.4347 (14)	C18—H18A	0.9900
C3—H3A	0.9900	C18—H18B	0.9900
C3—H3B	0.9900	C19—C20	1.5281 (16)
O4—C5	1.4344 (14)	C19—H19A	0.9900
C5—C6	1.5196 (18)	C19—H19B	0.9900
C5—H5A	0.9900	C20—H20A	0.9900
C5—H5B	0.9900	C20—H20B	0.9900
C6—N7	1.4677 (15)	C21—O22	1.2272 (14)
C6—H6A	0.9900	C21—C23	1.5314 (15)
C6—H6B	0.9900	C23—O24	1.4178 (13)
N7—C26^i^	1.3473 (15)	C23—H23A	0.9900
N7—C8	1.4695 (14)	C23—H23B	0.9900
C8—C9	1.5254 (16)	O24—C25	1.4202 (13)
C8—H8A	0.9900	C25—C26	1.5216 (16)
C8—H8B	0.9900	C25—H25A	0.9900
C9—C10	1.5153 (16)	C25—H25B	0.9900
C9—H9A	0.9900	C26—O27	1.2321 (14)
C9—H9B	0.9900	C26—N7^i^	1.3473 (15)
C10—O11	1.4270 (14)	C1S—C2S	1.3879 (18)
C10—H10A	0.9900	C1S—C6S	1.3910 (19)
C10—H10B	0.9900	C1S—H1S	0.9500
O11—C12	1.4271 (13)	C2S—C3S	1.3884 (18)
C12—C13	1.5030 (17)	C2S—H2S	0.9500
C12—H12A	0.9900	C3S—C4S	1.3842 (19)
C12—H12B	0.9900	C3S—H3S	0.9500
C13—O14	1.4257 (15)	C4S—C5S	1.3856 (18)
C13—H13A	0.9900	C4S—H4S	0.9500
C13—H13B	0.9900	C5S—C6S	1.3848 (19)
O14—C15	1.4269 (14)	C5S—H5S	0.9500
C15—C16	1.5066 (18)	C6S—H6S	0.9500
C15—H15A	0.9900		
			
C21—N1—C2	117.64 (9)	O14—C15—H15B	108.9
C21—N1—C20	124.81 (9)	C16—C15—H15B	108.9
C2—N1—C20	117.31 (9)	H15A—C15—H15B	107.7
N1—C2—C3	113.65 (9)	O17—C16—C15	108.46 (10)
N1—C2—H2A	108.8	O17—C16—H16A	110.0
C3—C2—H2A	108.8	C15—C16—H16A	110.0
N1—C2—H2B	108.8	O17—C16—H16B	110.0
C3—C2—H2B	108.8	C15—C16—H16B	110.0
H2A—C2—H2B	107.7	H16A—C16—H16B	108.4
O4—C3—C2	109.78 (9)	C18—O17—C16	113.39 (9)
O4—C3—H3A	109.7	O17—C18—C19	112.94 (10)
C2—C3—H3A	109.7	O17—C18—H18A	109.0
O4—C3—H3B	109.7	C19—C18—H18A	109.0
C2—C3—H3B	109.7	O17—C18—H18B	109.0
H3A—C3—H3B	108.2	C19—C18—H18B	109.0
C5—O4—C3	112.59 (8)	H18A—C18—H18B	107.8
O4—C5—C6	112.06 (9)	C18—C19—C20	114.43 (10)
O4—C5—H5A	109.2	C18—C19—H19A	108.7
C6—C5—H5A	109.2	C20—C19—H19A	108.7
O4—C5—H5B	109.2	C18—C19—H19B	108.7
C6—C5—H5B	109.2	C20—C19—H19B	108.7
H5A—C5—H5B	107.9	H19A—C19—H19B	107.6
N7—C6—C5	113.36 (10)	N1—C20—C19	113.30 (10)
N7—C6—H6A	108.9	N1—C20—H20A	108.9
C5—C6—H6A	108.9	C19—C20—H20A	108.9
N7—C6—H6B	108.9	N1—C20—H20B	108.9
C5—C6—H6B	108.9	C19—C20—H20B	108.9
H6A—C6—H6B	107.7	H20A—C20—H20B	107.7
C26^i^—N7—C6	117.95 (9)	O22—C21—N1	122.34 (10)
C26^i^—N7—C8	124.07 (9)	O22—C21—C23	120.75 (10)
C6—N7—C8	117.93 (9)	N1—C21—C23	116.91 (9)
N7—C8—C9	112.88 (9)	O24—C23—C21	111.41 (9)
N7—C8—H8A	109.0	O24—C23—H23A	109.3
C9—C8—H8A	109.0	C21—C23—H23A	109.3
N7—C8—H8B	109.0	O24—C23—H23B	109.3
C9—C8—H8B	109.0	C21—C23—H23B	109.3
H8A—C8—H8B	107.8	H23A—C23—H23B	108.0
C10—C9—C8	111.23 (9)	C23—O24—C25	112.15 (8)
C10—C9—H9A	109.4	O24—C25—C26	107.45 (9)
C8—C9—H9A	109.4	O24—C25—H25A	110.2
C10—C9—H9B	109.4	C26—C25—H25A	110.2
C8—C9—H9B	109.4	O24—C25—H25B	110.2
H9A—C9—H9B	108.0	C26—C25—H25B	110.2
O11—C10—C9	109.22 (9)	H25A—C25—H25B	108.5
O11—C10—H10A	109.8	O27—C26—N7^i^	122.37 (10)
C9—C10—H10A	109.8	O27—C26—C25	120.34 (10)
O11—C10—H10B	109.8	N7^i^—C26—C25	117.29 (10)
C9—C10—H10B	109.8	C2S—C1S—C6S	119.43 (12)
H10A—C10—H10B	108.3	C2S—C1S—H1S	120.3
C10—O11—C12	111.42 (9)	C6S—C1S—H1S	120.3
O11—C12—C13	110.40 (10)	C1S—C2S—C3S	119.94 (12)
O11—C12—H12A	109.6	C1S—C2S—H2S	120.0
C13—C12—H12A	109.6	C3S—C2S—H2S	120.0
O11—C12—H12B	109.6	C4S—C3S—C2S	120.43 (12)
C13—C12—H12B	109.6	C4S—C3S—H3S	119.8
H12A—C12—H12B	108.1	C2S—C3S—H3S	119.8
O14—C13—C12	109.13 (10)	C3S—C4S—C5S	119.77 (12)
O14—C13—H13A	109.9	C3S—C4S—H4S	120.1
C12—C13—H13A	109.9	C5S—C4S—H4S	120.1
O14—C13—H13B	109.9	C6S—C5S—C4S	119.96 (12)
C12—C13—H13B	109.9	C6S—C5S—H5S	120.0
H13A—C13—H13B	108.3	C4S—C5S—H5S	120.0
C13—O14—C15	113.38 (9)	C5S—C6S—C1S	120.47 (12)
O14—C15—C16	113.48 (10)	C5S—C6S—H6S	119.8
O14—C15—H15A	108.9	C1S—C6S—H6S	119.8
C16—C15—H15A	108.9		
			
C21—N1—C2—C3	92.67 (12)	O17—C18—C19—C20	−50.94 (14)
C20—N1—C2—C3	−81.95 (12)	C21—N1—C20—C19	115.78 (12)
N1—C2—C3—O4	73.21 (12)	C2—N1—C20—C19	−70.02 (12)
C2—C3—O4—C5	−178.20 (9)	C18—C19—C20—N1	−61.35 (13)
C3—O4—C5—C6	100.21 (11)	C2—N1—C21—O22	6.07 (15)
O4—C5—C6—N7	−59.09 (13)	C20—N1—C21—O22	−179.75 (10)
C5—C6—N7—C26^i^	−75.20 (13)	C2—N1—C21—C23	−173.17 (9)
C5—C6—N7—C8	102.34 (11)	C20—N1—C21—C23	1.00 (15)
C26^i^—N7—C8—C9	−102.18 (12)	O22—C21—C23—O24	10.07 (15)
C6—N7—C8—C9	80.43 (12)	N1—C21—C23—O24	−170.67 (9)
N7—C8—C9—C10	177.10 (9)	C21—C23—O24—C25	75.22 (11)
C8—C9—C10—O11	57.98 (13)	C23—O24—C25—C26	172.99 (8)
C9—C10—O11—C12	162.03 (9)	O24—C25—C26—O27	−110.47 (11)
C10—O11—C12—C13	169.66 (10)	O24—C25—C26—N7^i^	68.94 (12)
O11—C12—C13—O14	−83.13 (12)	C6S—C1S—C2S—C3S	1.05 (19)
C12—C13—O14—C15	−168.70 (9)	C1S—C2S—C3S—C4S	−0.38 (19)
C13—O14—C15—C16	−82.55 (13)	C2S—C3S—C4S—C5S	−0.64 (18)
O14—C15—C16—O17	−65.05 (13)	C3S—C4S—C5S—C6S	0.98 (18)
C15—C16—O17—C18	159.12 (10)	C4S—C5S—C6S—C1S	−0.31 (19)
C16—O17—C18—C19	−71.92 (12)	C2S—C1S—C6S—C5S	−0.71 (19)

Symmetry codes: (i) −*x*+1, −*y*+1, −*z*+1.

## References

[bb1] Bencini, A., Bianchi, A., Ciampolini, M., Dapporto, P., Fusi, V., Micheloni, M., Nardi, N., Paoli, P. & Valtancoli, B. (1992). *J. Chem. Soc. Dalton Trans.* pp. 2049–2054.

[bb2] Bruker (1998). *SMART* and *SAINT* Bruker AXS Inc., Madison, Wisconsin, USA.

[bb3] Cheetham, G. M. T. & Harding, M. M. (1991). *Acta Cryst.* C**47**, 2478–2479.

[bb4] Cheney, J., Kintzinger, J. P. & Lehn, J. M. (1978). *Nouv. J. Chim.* **2**, 411–418.

[bb5] Dietrich, B., Lehn, J. M., Sauvage, J. P. & Blanzat, J. (1973). *Tetrahedron*, **29**, 1629–1645.

[bb6] Fischer, J., Mellinger, M. & Weiss, R. (1977). *Inorg. Chim. Acta*, **21**, 259–263.

[bb7] Groth, P. (1986). *Acta Chem. Scand. Ser. A*, **40**, 154–156.

[bb8] Krakowiak, K. E., Bradshaw, J. S., Kou, X. & Dalley, N. K. (1995). *J. Heterocycl. Chem.* **32**, 931–935.

[bb9] Lehn, J. M. (1973). *Struct. Bond.* **16**, 1–69.

[bb10] Lehn, J. M. (1988). *Angew. Chem. Int. Ed. Engl.* **27**, 89–112.

[bb11] Lehn, J. M., Simon, J. & Wagner, J. (1977). *Nouv. J. Chim.* **1**, 77–84.

[bb12] Pascard, C., Riche, C., Cesario, M., Kotzyba-Hibert, F. & Lehn, J. M. (1982). *J. Chem. Soc. Chem. Commun.* pp. 557–560.

[bb13] Plenio, H. & Diodone, R. (1995). *J. Organomet. Chem.* **492**, 73–80.

[bb14] Rebizant, J., Spirlet, M. R., Barthelemy, P. & Desreux, J. F. (1984). *Acta Cryst.* C**40**, 484–486.

[bb15] Sheldrick, G. M. (2007). *SADABS* University of Göttingen, Germany.

[bb16] Sheldrick, G. M. (2008). *Acta Cryst.* A**64**, 112–122. 10.1107/S0108767307043930 18156677

[bb17] Smith, G. L. N., Rocher, N. M., Powell, D. R. & Taylor, R. W. (2007). *Acta Cryst.* E**63**, o1253–o1255.

[bb18] Wiest, R. & Weiss, R. (1973). *J. Chem. Soc. Chem. Commun.* pp. 678–679.

